# Chaperone-usher fimbriae in a diverse selection of *Gallibacterium* genomes

**DOI:** 10.1186/1471-2164-15-1093

**Published:** 2014-12-12

**Authors:** Eglė Kudirkienė, Ragnhild J Bager, Timothy J Johnson, Anders M Bojesen

**Affiliations:** Department of Veterinary Disease Biology, University of Copenhagen, Stigbøjlen 4, 1870 Frederiksberg C, Denmark; Department of Veterinary and Biomedical Sciences, University of Minnesota, Saint Paul, Minnesota USA

## Abstract

**Background:**

Fimbriae are bacterial cell surface organelles involved in the pathogenesis of many bacterial species, including *Gallibacterium anatis,* in which a F17-like fimbriae of the chaperone-usher (CU) family was recently shown to be an important virulence factor and vaccine candidate. To reveal the distribution and variability of CU fimbriae 22 genomes of the avian host-restricted bacteria *Gallibacterium* spp. were investigated. Fimbrial clusters were classified using phylogeny-based and conserved domain (CD) distribution-based approaches. To characterize the fimbriae in depth evolutionary analysis and *in vitro* expression of the most prevalent fimbrial clusters was performed.

**Results:**

Overall 48 CU fimbriae were identified in the genomes of the examined *Gallibacterium* isolates. All fimbriae were assigned to γ4 clade of the CU fimbriae of Gram-negative bacteria and were organized in four-gene clusters encoding a putative major fimbrial subunit, a chaperone, an usher and a fimbrial adhesin. Five fimbrial clusters (Flf-Flf4) and eight conserved domain groups were defined to accommodate the identified fimbriae. Although, the number of different fimbrial clusters in individual *Gallibacterium* genomes was low, there was substantial amino acid sequence variability in the major fimbrial subunit and the adhesin proteins. The distribution of CDs among fimbrial clusters, analysis of their flanking regions, and evolutionary comparison of the strains revealed that *Gallibacterium* fimbrial clusters likely underwent evolutionary divergence resulting in highly host adapted and antigenically variable fimbriae. *In vitro*, only the fimbrial subunit FlfA was expressed in most *Gallibacterium* strains encoding this protein. The absence or scarce expression of the two other common fimbrial subunits (Flf1A and Flf3A) indicates that their expression may require other *in vitro* or *in vivo* conditions.

**Conclusions:**

This is the first approach establishing a systematic fimbria classification system within *Gallibacterium spp.*, which indicates a species-wide distribution of γ_4_ CU fimbriae among a diverse collection of *Gallibacterium* isolates. The expression of only one out of up to three fimbriae present in the individual genomes *in vitro* suggests that fimbriae expression in *Gallibacterium* is highly regulated. This information is important for future attempts to understand the role of *Gallibacterium* fimbriae in pathogenesis, and may prove useful for improved control of *Gallibacterium* infections in chickens.

**Electronic supplementary material:**

The online version of this article (doi:10.1186/1471-2164-15-1093) contains supplementary material, which is available to authorized users.

## Background

*Gallibacterium* is among the most important bacteria infecting the reproductive organs of laying hens [1, 2]. The bacterium often constitutes a part of the normal microflora in the upper respiratory and lower reproductive tract of chickens, however under certain conditions it causes lesions such as peritonitis and salpingitis. Following infection, egg production is significantly reduced and infected birds may also be more susceptible to other infections [3–6]. Organisms within the genus *Gallibacterium* appear to be restricted to avian hosts, including both domesticated and wild bird species [7–9]; however, there are three closely related species including *Gallibacterium anatis*, *Gallibacterium* genomospecies 1 and *Gallibacterium* genomospecies 2 that mainly affect chickens [8]. All three species are strongly haemolytic (except *G. anatis* bv. *anatis*) due to the expression of a RTX-toxin GtxA, which is an important and well-described virulence factor of *Gallibacterium* [10, 11]. Several other virulence-associated factors including a polysaccharide capsule [2], secreted metalloproteases [12] and several fimbriae have been identified in *Gallibacterium* [13, 14]. Among these, the role of one fimbrial type in the pathogenesis of *Gallibacterium anatis* was recently investigated. A 1 to 2 μm -long F17-like fimbriae on the surface of *Gallibacterium anatis* bv. *haemolytica* strain 12656–12 was initially described by Bager et al. [15]. The fimbria is encoded by the four-gene fimbrial cluster *flf* and assembled via the chaperone-usher (CU) pathway. *In vitro* studies using chicken epithelial cells as well as animal infection experiments demonstrated that this fimbrium might be involved in *G. anatis* colonization of the upper respiratory tract [13] and play an important role in the pathogenesis in chickens [15]. Whole genome sequencing of *Gallibacterium* strains has revealed the presence of at least two homologous *flf* fimbrial clusters in several *Gallibacterium* genomes [14], however the function and role of these fimbriae in the pathogenesis of *Gallibacterium* is yet unknown.

Fimbriae are surface organelles expressed by many commensal and pathogenic bacteria. They are often involved in the adhesion of the bacteria to epithelial and/or sub-epithelial surfaces of various tissues [16–27]. Among a wide range of fimbriae expressed by Gram-negative bacteria the CU type fimbriae are the most prevalent [28] and the presence of up to 17 fimbrial clusters of this type in a single bacterial genome have previously been reported [27]. Most of the CU fimbriae are composed of the four structural genes; a major fimbrial subunit, a chaperone, an usher and a tip adhesin. However some CU fimbrial clusters may contain additional genes encoding structural, assembly or regulatory proteins [28, 29]. Different studies have showed that the tip adhesin encodes various receptors recognizing specific ligands in the host tissues. Consequently the interaction of the CU fimbriae with the host is highly specific, and the expression of different fimbriae may be associated with different stages and sites of the infection [19, 30, 31]. To be able to determine the evolutionary and functional relationship between different CU fimbriae present in Gram-negative bacteria, they were classified into seven phylogenetic clades, each including fimbriae sharing conserved domains in the major fimbrial subunit and the tip adhesin proteins with common characteristics [28]. Fimbriae have been intensively studied, not only because they are important virulence factors of bacteria, but also because they are among the most widely used targets for the development of interventions such as vaccines [25, 31–38]. Recently, the *Gallibacterium* F17-like fimbrial subunit protein (FlfA) was identified as a promising candidate that may be used to vaccinate laying hens [39].

This study aimed to analyze the distribution and variation of CU fimbrial clusters in the genomes of a selection of genetically diverse *Gallibacterium* strains, and to characterize their expression levels during *in vitro* conditions. Detailed phylogenetic analysis of the genes encoding fimbrial proteins allowed the development of a fimbrial classification system within *Gallibacterium* based on the evolutionary relationship between different fimbrial types.

## Results

### Identification of chaperone-usher type fimbrial clusters

A total of 22 strains representing three *Gallibacterium* species (*G. anatis*, *G*. genomospecies 1 and *G*. genomospecies 2) frequently associated with the reproductive tract infection of laying hens were selected for the study, aimed at broad genotypic representation within the species investigated (Table 1). A bioinformatic analysis of the *Gallibacterium* genomes revealed 48 intact four-gene CU clusters in total. All clusters encoded a major fimbrial subunit, a chaperone, an usher and a fimbrial adhesion protein. The number of fimbrial clusters varied between strains with three fimbrial clusters detected in the genomes of eight of the strains, two clusters in 11 strains, and one cluster in two strains [see Additional file 1: Table S1]. One strain (20558/3kl.) did not appear to have any fimbrial assembly gene in its genome.

**Table 1:** **Strains used in the study**

Species	Strain name_alternative name	Host/lessions_country_year ^a^
*G. anatis* biovar *anatis*	F149_ATCC43329^T^	Duck/Intestine_DK_1979
*G. anatis* biovar *haemolytica*	23 K10	Chicken/no lession_DK
	23 T10	Chicken_DK
	21 K2	Chicken_DK
	21 T2	Chicken_DK
	36961/Sv7	Chicken_DK
	10672-6 Salp	Chicken/Salpingitis_DK
	12158-5 Salp	Chicken_DK
	10672/9 Salp_F114	Chicken_GER
	12656-12 Liver	Chicken/Liver_DK_1981
	F0003406	Turkey/Liver_USA
	1797	Turkey/Joint_GER
	7990	Chicken_MEX
	4895	Chicken_MEX
	Avicor	Chicken_MEX
	IPDH697-78	Chicken_GER_1978
	Gerl. 3348/80_F219	Goose_GER
	20558/3kl.	Goose_DK
	CCM5995_Mraz26b/65A	Chicken_CZ_1978
	Gerl.4224/88	Avian_GER
*G. genomospecies* 1	CCM5974_Mraz556/71B	Chicken/Liver_CZ
*G. genomospecies* 2	CCM5976_Mraz576a/71D	Chicken/Oviduct_CZ

^a^Information on the origin of the strains was obtained from Bojesen et al. [1, 40], Christensen et al. [8], Bojesen et al. [2], Bojesen and Shivaprasad [7], Bisgaard et al. [9], Kristensen et al. [11].

### Phylogenetic analysis of *Gallibacterium*CU fimbrial clusters

**Figure 1:**
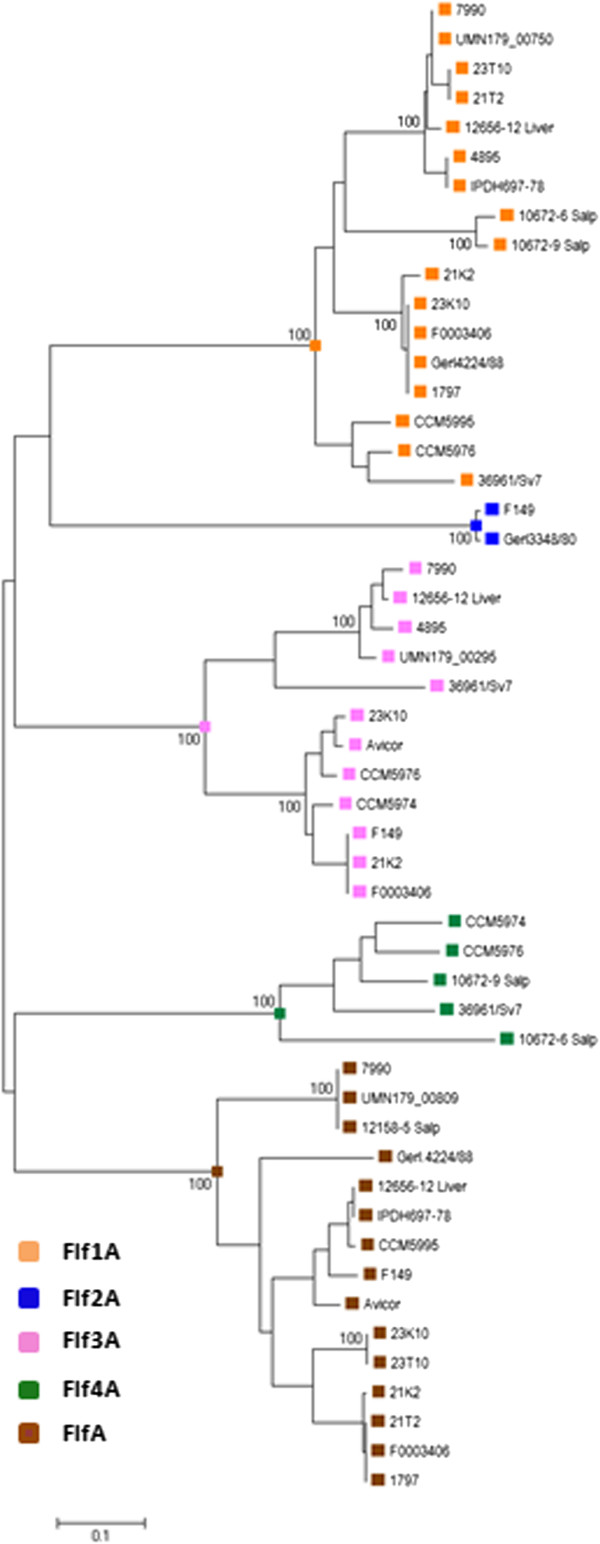
**Evolutionary relationships of fimbrial subunit proteins identified in** ***Gallibacterium*** **strains.** A total of 151 amino acid positions were used to infer the evolutionary relationship of 51 aligned fimbrial subunit proteins. Data was analyzed using the Neighbor-Joining method and conducted in MEGA6. Bootstrap values (1000) of more than 90 are displayed next to the branches. The scale represents the number of amino acid substitutions per site computed using the Poisson correction method. Fimbrial subunit proteins were classified into 5 phylogenetically distinct groups (FlfA, Flf1A, Flf2A, Flf3A and Flf4A) shown in different colors.

To reveal the phylogenetic relationship between the fimbrial clusters, individual phylogenetic trees for each of the fimbrial assembly protein were constructed. This revealed three distinct phylogenetic groups based on the chaperone, usher and adhesin sequences, respectively [see Additional file 2: Figure S1, Additional file 3: Figure S2, Additional file 4: Figure-S3], and five groups based on the fimbrial subunit protein sequences (Figure 1).

To represent possible antigenic variation between *Gallibacterium* fimbrial clusters, all *Gallibacterium* CU fimbriae were classified into five types based on fimbrial subunit phylogeny. All fimbrial clusters in which a previously described FlfA [15] homolog was detected were named as Flf, and those in which major fimbrial subunits were assigned to the different phylogenetic groups than FlfA were named as Flf1, Flf2, Flf3 and Flf4. Out of the five defined CU fimbrial clusters, the most common fimbrial cluster was Flf1 found in 74% *Gallibacterium* genomes, followed by Flf and Flf3 detected in 65% and 52% of the genomes, respectively.

### Pair-wise comparison of CU fimbrial proteins

A pair-wise comparison of the amino acid sequences of all proteins in the CU clusters was performed to determine the similarity between the fimbrial proteins assigned to the different fimbrial types. The protein similarity within the phylogenetic groups was ≥80.3%, ≥70.2% and ≥66.7% for the chaperones, ushers and the major fimbrial subunits, respectively, whereas the adhesins were much more variable with similarities ≥16.2% found between adhesins belonging to the same phylogenetic group. Fimbrial subunits belonging to different phylogenetic groups were 30.5% to 50.8% similar, chaperones 52.9% to 68.5%, ushers 44.0% to 71.4%, and adhesins 9.7% to 24.2% similar, respectively. The analysis revealed that both within and between the phylogenetic groups, the fimbrial adhesins show the most variable amino acid sequences in comparison to the sequences of the major fimbrial subunit, the chaperone and the usher.

### Analysis of the regions flanking CU clusters

Analysis of the flanking regions of the fimbrial clusters was employed to identify possible homology between different CU clusters found in *Gallibacterium* spp. and the grouping based on major fimbrial subunit phylogeny. Most of the CU clusters assigned to the different fimbrial types were flanked by non-homologous genes (see Additional file 5: Table S2). However there were some exceptions to this observation. The *flf* and *flf4* CU fimbrial clusters were surrounded by homologous genes. An additional exception was found for the fimbriae of strain 4895. Although the CU cluster found in this strain was assigned to fimbrial clusters *flf3*, it was flanked with genes homologous to the genes flanking the fimbrial cluster *flf*. This may suggest the common ancestry of the *flf3* and the *flf* fimbriae. Interestingly, the genes flanking CU clusters were found in all analyzed *Gallibacterium* genomes independently from the presence or absence of the CU clusters in the genome. In some cases other genes were found inserted instead of the CU cluster (data not shown). Further flanking region analysis revealed the presence of mobile elements and transposases upstream or downstream of the CU clusters. The mobile elements were found upstream the major fimbrial subunit gene *flf3A* in 10 out of the 12 strains harboring the *flf3* cluster. Such mobile elements were not detected in strain 4895, whereas in strain F000340, a mobile element downstream the gene encoding the fimbrial adhesin was detected. A lower prevalence of mobile elements was found around the fimbrial cluster *flf*. Only two strains (12158–5 and UMN179) belonging to this fimbrial type had mobile elements present upstream the major fimbrial subunit gene *flfA*. No mobile elements were detected in the flanking regions of CU clusters belonging to the other fimbrial subunit phylogenetic groups.

The widespread presence of mobile elements and transposases in the regions flanking the CU clusters supports previous suggestions that fimbriae may be horizontally transferred within *G. anatis* [14]. Additionally, the high similarity of the flanking regions virtually in all *Gallibacterium* genomes indicates that homologous recombination likely is involved in the exchange of the fimbriae between closely related strains of *G. anatis* [41].

### Conserved domain identification in CU fimbrial cluster proteins

**Figure 2:**
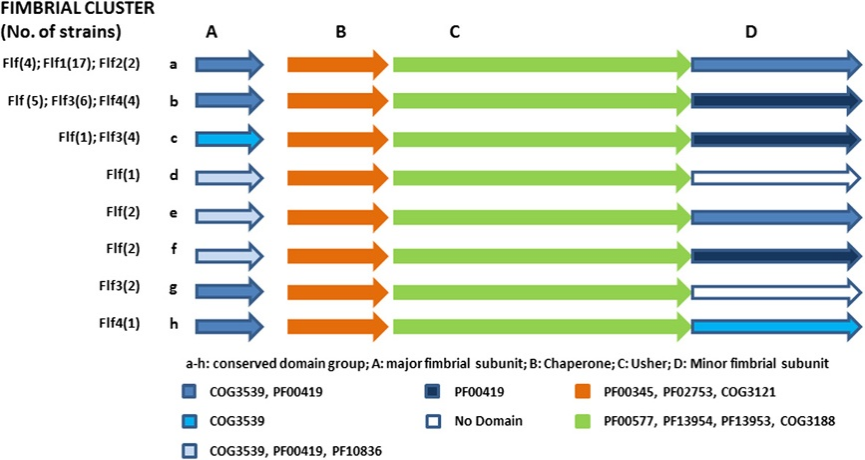
**Conserved domains (CD’s) groups identified among 48 CU clusters present in 22** ***Gallibacteriums*** **strains including UMN179.** Letters from A-D indicate the major fimbrial subunit, the chaperone, the usher and the minor fimbrial subunit proteins, respectively. Letters from a to h are used to classify fimbrial clusters based on different CD’s combinations found within the fimbrial assembly proteins in the cluster. In the figure CD’s with the reported value of p <0.01 are shown.

The presence or absence of a particular conserved domain within the proteins investigated may indicate evolutionary relationships and functional similarities between the fimbrial proteins. Therefore, and to further compare the fimbrial clusters of *Gallibacterium*, the conserved domains (CD) in the proteins forming CU clusters were identified. This analysis revealed that the same CD’s were present in all the chaperones and the ushers belonging to different CU clusters of *Gallibacterium* spp. (Figure 2). However different combinations of the three CDs (COG3539, PF00419 and PF10836) within the major fimbrial subunit proteins, as well as the three CDs (COG3539, PF00419 and COG4889) within the fimbrial adhesin proteins belonging to different CU clusters were observed. The presence or absence of CDs in these proteins did not correlate with the fimbrial subunit or adhesin phylogenies. Based on CD variation, all CU clusters could be classified into eight CD groups named “a” to “h” (Figure 2). Among these, the “a” and “b” groups were found to be the most prevalent and represented in 43.4% and 28.3% of the CU clusters, respectively. The CDs in the Flf1 cluster were highly similar and assigned to the CD group “a”. Conversely, different CD combinations were found both in the fimbrial subunits and the adhesins of the fimbrial cluster Flf. In total, six different CD combinations were detected in the fimbrial cluster Flf, of which the CD groups “a” and “b” were found to be the most commonly present (26.7% and 33.3%, respectively). The remaining Flf fimbriae were assigned to several other CD groups defined in the study. For Flf3, the highest proportion of fimbriae was assigned to CD groups “b” and “c”. The CD groups found in the four remaining fimbrial clusters are shown in Figure 2.

This analysis showed that different combinations of CDs are present in the proteins encoded by fimbrial clusters of *Gallibacterium*. CD identification in the CU clusters would enable future insight into the evolutionary and functional relationships of *Gallibacterium* CU fimbriae with fimbriae from other Gram-negative bacteria and fimbriae in uncharacterized *Gallibacterium* isolates.

### *Gallibacterium*CU fimbriae similarity to the fimbriae of other bacterium species

To reveal the evolutionary relationship of *Gallibacterium* fimbriae to the fimbriae of other Gram-negative bacterial species protein sequences of all 48 ushers identified in this study were compared to the usher protein sequences used by Nuccio and Bäumler [28]. Based on this analysis, all *Gallibacterium* fimbriae were assigned to Nuccio clade γ and more specifically clustered together with fimbrial ushers of γ_4_ sub-clade [see Additional file 6: Figure S4].

The dominating cluster assemblies of *Gallibacterium* spp. were compared to the assemblies of fimbriae found in *E. coli*. The same structural proteins and CDs as in *Gallibacterium* CD class “a” was found in F17 and same as “b” in Yeh-like fimbriae of *E.coli*, both belonging to γ_4_ Nuccio clade [26].

The BLASTp of amino acid sequences against UniProt/SwissProt database revealed that the major fimbrial subunits of the CU clusters Flf, Flf1, Flf2 and Flf3 were most similar (from 32% to 45%, from p = 1e^-11^ to p = 6e^-34^, depending on the strain) to the F17 fimbrial subunit of *E. coli* [GenBank: P11312.2]. However, fimbrial subunits of the fimbrial cluster Flf4 were most similar (from 30% to 35%, from p = 4e^-13^ to p = 1e^-19^, depending on the strain) to FimA fimbrial subunit protein of *Salmonella enterica* serovar Tyhimurium [GenBank: P37921].

To further examine the relationships between *Gallibacterium* CU fimbriae and fimbriae of other bacteria, BLASTp analysis against the NCBI non-redundant protein database was performed. This analysis showed that the ushers and chaperones of the CU fimbriae Flf, Flf1, Flf3 and Flf4 were most similar to those found in *Gallibacterium* strain UMN179 and *Avibacterium paragallinarum*. The CU fimbriae Flf2 were most similar to the chaperones and ushers present in F17-like fimbriae of uropathogenic *E. coli*. The major fimbrial subunit proteins and adhesins of the CU fimbriae Flf, Flf3 and Flf4 were most similar to the proteins found in *Gallibacterium* strain UMN179 and *Avibacterium paragallinarum*. However, Flf1A and Flf2A homologs were detected in *Acinetobacter baumannii*, uropathogenic *Proteus mirabilis* and *Esherichia coli*, *Haemophilus influenza* and *Haemophilus aegypticus* [see Additional file 7: Table S3].

In conclusion, the proteins of the *Gallibacterium* CU clusters Flf, Flf3 and Flf4 are most similar to each other, suggesting that these clusters may have evolved from a common ancestor or have arisen due to gene duplication. Moreover, the presence of a fimbrial cluster identical to the *Gallibacterium* FlfA in the genome of *A. paragallinarum* suggest that this fimbriae may have been recently exchanged between these two species, which could theoretically occur during natural co-colonization of the chicken upper respiratory tract.

### Distribution of different CU fimbrial clusters among MLST types of *G. anatis*and *G.*genomospecies 1 and 2

**Figure 3:**
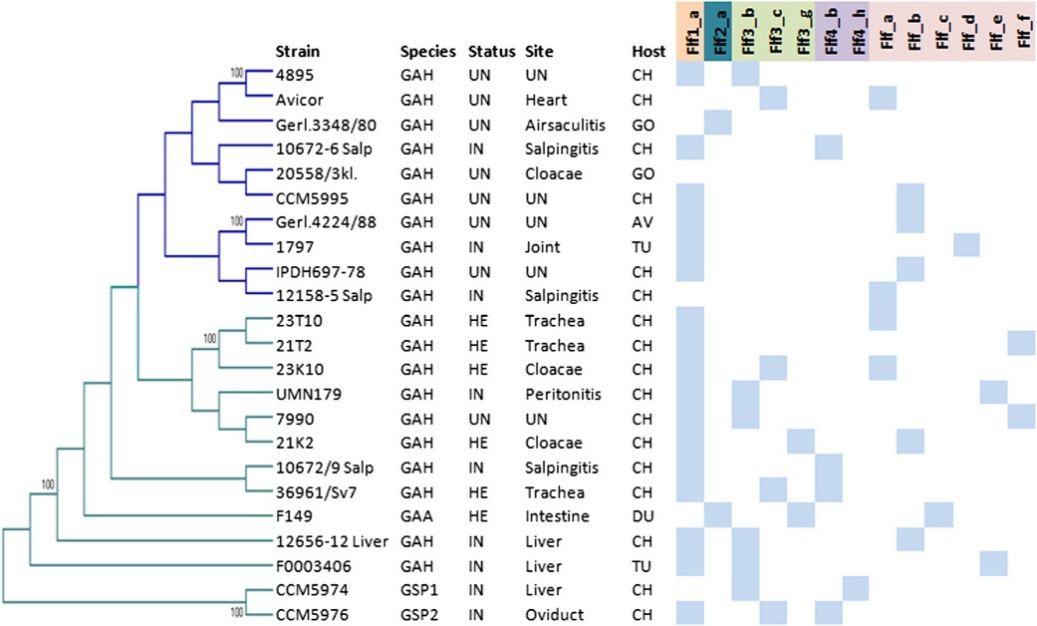
**Distribution of 13 fimbrial types among** ***G. anatis*** **and** ***G. genomospecies*** **1 and 2 strains in an evolutionary context.** The tree presented in the left was constructed using concatenated sequences of 8 housekeeping genes present in 23 *Gallibacterium* genomes. There were a total of 4176 positions in the final dataset and 1000 bootstrap replicates were included. The evolutionary history was inferred by using the Maximum Likelihood method based on the Hasegawa-Kishino-Yano model in MEGA6. Light blue cells on the right represent the distribution of 13 fimbrial types determined based on fimbrial subunit phylogeny (FlfA-FlfA4) and presence/absence of CD’s in fimbrial clusters among the strains. Depending on the distribution of different fimbrial types the tree is divided into the blue and green branches, where strains at the ends of green branches maintain higher variation of fimbrial types.

The fimbrial subunit phylogeny-based and the CD-based grouping of *G. anatis* and *G. genomospecies* 1 and 2 CU clusters defined in this study (Figures 1 and 2) were combined with, and correlated to the phylogenetic outline provided by multi-locus sequence typing. This depicted 13 different fimbrial types in total. Each fimbrial type was named according to the corresponding fimbrial subunit phylogenetic group and CD group as described previously. The distribution of the different fimbrial types among the 22 *Gallibacterium* strains (including UMN179), which were isolated from different bird species, body sites, and countries, were clustered using eight concatenated sequences of housekeeping genes of *Gallibacterium* [14]. The comparisons are summarized in Figure 3. No correlation between the distribution of different fimbrial types and the origin of isolation was observed. However, a difference in the fimbrial type occurrence among closely related (blue branches) and distant *Gallibacterium* strains (green branches) as shown in the MLST tree was detected. Moreover, a higher fimbrial variation was found among distantly related strains including *G.* genomospecies 1 and *G.* genomospecies 2. Nine out of the 13 strains had three fimbrial types encoded in their genomes with the fimbrial clusters Flf3 and Flf4 being the most common source of variation. Additionally, these strains contained seven of the CD groups found within the CU fimbrial clusters, whereas more closely related strains at the end of the blue branches, typically only encoded only one or two fimbrial types, and only three different CDs groups. This analysis revealed that Flf, Flf1 and Flf3 appear to be core fimbrial types that may be transferred between different ST types of *G. anatis*, and in case of Flf1 and Flf3, also between closely related species of *Gallibacterium* spp.

### *In vitro*expression of FlfA, Flf1A and Flf3A

**Figure 4:**
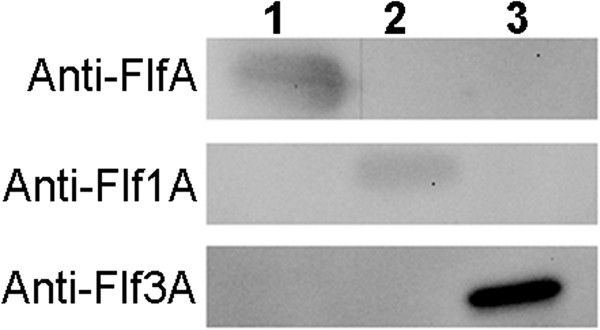
**Cross-reactivity of polyclonal antiserum raised against recombinant fimbrial subunit proteins.** The specificity of the polyclonal antiserum raised against FlfA, Flf1A and Flf3A was demonstrated by Western blotting comparing recognition of recombinant proteins by rabbit anti-FlfA, anti-Flf1A or anti-Flf3A antiserum. 0.1 μg of each recombinant protein was loaded. Lane 1, FlfA; lane 2, Flf1A; lane 3; Flf3A.

**Figure 5:**
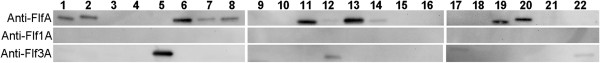
***In vitro*** **expressions of F17-like fimbrial subunit proteins.** The *in vitro* expression of FlfA, Flf1A and Flf3A was analyzed by Western blotting using immune rabbit antisera. Whole-cell extract equivalent to an OD_260_ of 8 was loaded to each lane. Lane 1, *G. anatis* 7990; Lane 2, *G. anatis* IPDH 697–78; Lane 3, *G. anatis* F149; Lane 4, *G. anatis* 10672*–*6 Salp; Lane 5, *G. anatis* 4895; Lane 6, *G. anatis* CCM5995; Lane 7, *G. anatis* 12656*–*12 Liver; Lane 8, *G. anatis* Avicor; Lane 9, *G. anatis* 10672/9 Salp; Lane 10, *G. anatis* 12158–5 Salp; Lane 11, *G. anatis* 21 T2; Lane 12, *G. anatis* 23 K10; Lane 13, *G. anatis* 23 T10; Lane 14, *G. anatis* 21 K2; Lane 15, *G. anatis* 36961/Sv7; Lane 16, *G. anatis* 20558/3kl; Lane 17, *G.* genomospecies 1 CCM5974; Lane 18, *G*. genomospecies 2 CCM5976; Lane 19, *G. anatis*1797; Lane 20, *G. anatis* Gerl.4224/88; Lane 21, *G. anatis* Gerl.3348/80; Lane 22, *G. anatis* F0003406.

To facilitate an investigation of the *in vitro* expression levels of the three main fimbrial subunit types FlfA, Flf1A and Flf3A, polyclonal antiserum against recombinant fimbrial proteins was raised in rabbits. The specificity of the antiserum was verified by Western blotting, comparing pre-immune and immune antiserum recognition of recombinant protein (data not shown). Moreover, Western blotting showed that each antiserum did not cross-react with the other fimbrial subunits (Figure 4), indicating limited overlap between epitopes of each fimbrial subunit protein. A whole-cell extract was prepared from cells in the early-stationary growth phase of each of the 22 *Gallibacterium* strains included in the study, and analyzed by Western blotting using rabbit antiserum (Figure 5). We found that FlfA was expressed *in vitro* in 11 out of the 14 strains containing the fimbrial cluster *flf*. Seven *Gallibacterium* strains possessing the *flf* cluster also encoded the *flf3* cluster in their genomes. However, only one strain expressed Flf3A at the expense of FlfA, while one strain (23 K10) expressed both proteins under the conditions tested. Flf3A expression was observed in three additional strains that did not carry *flf*. Compared to FlfA only a few isolates (4/11) containing the fimbrial cluster *flf3* also expressed the major fimbrial subunit Flf3A. *Gallibacterium* strains that did not contain or expressed FlfA or Flf3A, carried the fimbrial clusters *flf1*, *flf2* or *flf4*, whereas one goose isolate (12158) did not appear to have any fimbrial clusters apart from *flf*. The fimbrial cluster *flf2* was present only in strains isolated from a duck (F149) and a goose (Gerl. 3348/80) and *flf4* fimbrial cluster was found only in five *Gallibacterium* genomes. Consequently, the expression of Flf2A and Flf4A was not examined in this study. No expression of Flf1A was detected in any of the strains investigated under the conditions tested. The specificity of the bands was confirmed by Western blotting using pre-immune sera (data not shown). Collectively, the results show that the widely distributed F17-like fimbriae FlfA also was expressed in most of the *Gallibacterium* strains tested in this study. On the other hand, expression of F17-like fimbriae composed by Flf3A and especially Flf1A might be dependent on conditions not accounted for in the current setup as no correlation was found between the presence of a gene cluster and *in vitro* expression.

## Discussion

Research on the chaperone – usher (CU) fimbriae of *Gallibacterium* spp. initially gained interest because they were found to be important virulence factors and possible vaccine candidates [13, 15, 39]. In the present study we aimed to examine the presence, diversity and *in vitro* expression levels of CU fimbriae in a diverse collection of *Gallibacterium* strains isolated from different sites in healthy and diseased birds of different geographical origin. We propose an expandable classification system of *Gallibacterium* CU fimbriae based on the phylogenetic- and CD-based approaches corresponding to the generic Gram-negative bacteria CU fimbriae classification system developed by Nuccio and Bäumler [28].

### The few types of CU fimbrial clusters encoded by *Gallibacterium*have a similar structure

Sequence analysis of the 22 genomes representing three *Gallibacterium* species commonly associated with the chicken host, enabled an identification of 48 chromosomally located CU fimbriae. All fimbrial clusters resembled the archetype structure of the fimbrial gene cluster [28], encoding four essential structural fimbrial proteins: the major fimbrial subunit, the chaperone, the usher and an adhesin. The order of the genes was congruent in all of the investigated genomes. The structure of the CU fimbriae in *Gallibacterium* corresponded to what has been described for the F17 and *yeh* fimbriae of *E. coli* [23, 26], as well as in *stc* and *sti* fimbriae of *S. enterica* serovar Typhimurium [18].

Multiple alignment and phylogenetic comparison of the individual proteins encoded by each of the fimbrial clusters revealed a higher variation among the major fimbrial subunits compared to the other structural proteins (Figure 1) [see Additional file 2: Figure S1, Additional file 3: Figure S2, Additional file 4: Figure S3]. Previous findings indicate that major fimbrial subunits are the primary antigenic determinants of the fimbriae [42] and the pattern of evolutionary divergence of their amino acid sequences strongly correlates with the antigenic properties of the fimbriae [34, 43]. Therefore fimbrial subunit proteins were used for the primary classification of the fimbrial clusters of *Gallibacterium* spp. Based on the fimbrial subunit phylogenies, all *Gallibacterium* fimbriae were classified into five types named Flf, Flf1, Flf2, Flf3 and Flf4. Three of these types (Flf, Flf1 and Flf3) constituted the majority of the fimbrial clusters found in the examined *Gallibacterium* genomes and were homologous to the previously reported fimbrial clusters of UMN179_809-812, UMN179_750-753 and UMN179_295-292, respectively [14]. In addition, two distinct, previously unreported fimbrial clusters were identified in several *Gallibacterium* genomes (Flf2 and Flf4). Interestingly, the Flf2 fimbrial type was detected only in two *Gallibacterium* strains (F149 and Gerl.3348/80), which were isolated from a duck and a goose, respectively. Considering that the Flf2 flanking regions were more widespread among the strains investigated than the Flf2 locus itself could suggest that this fimbrial locus may have been more prevalent and possibly have been lost during adaptation in a chicken host [44].

Frequently two to three different CU fimbrial clusters were identified in each individual *Gallibacterium* genome. In contrast, possession of up to 7, 16, 12 and 17 distinct CU fimbrial clusters have been reported from individual strains of *E. coli* O157:H7 [19, 26], *E. coli* K-12 [22], *S. enterica* serovar Typhimurium [18, 45] and *P. mirabilis*[27], respectively. In general, CU fimbriae belonging to evolutionary distinct clades as defined by Nuccio and Bäumler [28] can be simultaneously detected in the genomes of Gram-negative bacteria (24,26,27), whereas all the identified *Gallibacterium* fimbriae were assigned to a single γ4 clade. The extensive variability between fimbriae in other bacterial species, such as *P. mirabilis*, *Salmonella* and *E. coli* suggest that there may be an evolutionary pressure for these bacteria to maintain a large fimbrial arsenal. Common to all these species is that they are found in variety of ecological niches including different hosts, animals and humans, as well as in the environment, whereas *G. anatis* is known as avian restricted pathogen [8].

### High sequence variation in the major fimbrial subunit and in the adhesin proteins may be related to the antigenic variation

In comparison to other bacterial species [27, 30, 46, 47]*Gallibacterium* generally did not possess a broad range of different fimbrial clusters, yet high sequence diversity of the major fimbrial subunits and adhesins within each fimbrial type was observed. Both the major fimbrial subunit protein and the adhesin protein are exposed on the surface of the bacterial cell and therefore determine the antigenic characteristics of the fimbriae [32, 42]. High sequence diversity in these proteins is thus likely to be maintained to enable specific interaction with different oligosaccharide motifs or receptors in host tissues, as well as to allow escape from recognition of the host immune system [30, 43, 48, 49]. As a result, host immunity exerts a constant pressure shaping new antigenic variants evading acquired host immunity [50]. The antigenic variation of *Gallibacterium* fimbriae may be a result of homologous recombination as described for many virulence-associated factors including fimbrial proteins of the human restricted pathogen *N. meningitidis* [51]. All identified *Gallibacterium* fimbrial clusters were embedded in one of five regions flanked by genes conserved even in distantly related *Gallibacterium* genomes. Therefore, we suggest that homologous recombination of the fimbrial loci may be the most likely explanation of the observed sequence diversity and a likely cause of antigenic variation in *Gallibacterium* spp.

### *Gallibacterium*fimbriae may be transferred between *Gallibacterium*strains, species and closely related genera

According to the BLASTp analyses, fimbrial clusters Flf, Flf3 and Flf4 were phylogenetically related and most similar to each other as well as to the fimbriae of *A. paragallinarum*, whereas Flf1 and Flf2 shared close relatedness with the fimbrial proteins found in *A. baumanni*, *P. mirabilis*, *E. coli* and *Haemophilus* species [see Additional file 7: Table S3]. Interestingly, the Flf1 and Flf2 fimbrial clusters possessed a highly conserved domain structure, belonging to only one CD group (group ‘a’). The same domain structure was also present in the very well characterized F17 fimbriae of *E. coli* [26]. Even though similar domain structures were shared with the fimbriae of *E. coli* suggesting a common ancestry, *Gallibacterium* fimbriae may be functionally different [52]. Duplication of the fimbrial clusters Flf1 and Flf2 is very likely, as several *Gallibacterium* clusters of the Flf type also possessed the ‘a’ CD structure. The CD structure of the remaining Flf clusters was highly diverse and represented by seven (b-h) CD groups in total (Figure 2). Some of these CD groups were shared with the fimbrial types Flf3 and Flf4 suggesting that fimbrial cluster duplication in *Gallibacterium* may occur [24]. If this is the case fimbrial clusters maintaining different CD structure may encode functionally different fimbriae that may interact with different host tissues and the receptors. Furthermore, the change of the domain structure may induce altered pilus attachment properties or even fimbrial structure, which again could help *Gallibacterium* to evade the host immune response and improve its survival within the host [23, 53].

The comparison of *Gallibacterium* strains based on the sequence similarity of eight loci conserved in 22 *Gallibacterium* genomes [14] confirmed that all examined *Gallibacterium* strains were genetically diverse. Different MLST lineages shared fimbrial clusters belonging to different fimbrial types identified in *Gallibacterium* strains*.* We found no relationship between the presence of a specific fimbrial type or CD group and the health status or isolation site in the host, which may indicate that distinct fimbrial types may be equally present in different *Gallibacterium* strains, whereas their gene expression levels may be tightly regulated during the colonization process in the host [54–56]. The distribution of different fimbrial types in genetically distinct isolates of *Gallibacterium* strains indicates that horizontal transfer of the fimbrial clusters occurs between strains. Moreover, similar fimbrial types appear in closely related species *G. anatis*, *G.* genomospecies 1 and *G.* genomospecies 2 [see Additional file 8: Figure S5], which were all isolated from the chicken host suggesting a horizontal gene transfer between less-related strains of different *Gallibacterium* species. The presence of mobile genetic elements in close proximity of the fimbrial gene clusters further supports this notion. The major fimbrial subunit protein FlfA of *Gallibacterium* was highly similar (66-100%) to a protein from *Avibacterium paragallinarum* [see Additional file 9: Figure S6] indicating that lateral gene transfer even may take place between closely related genera [see Additional file 8: Figure S5]. *G. anatis* and *A. paragallinarum* share the upper respiratory tract of chickens as their main habitat [1, 57], thus it is very likely that Flf fimbriae has been exchanged between these two genera during co-colonization of this niche. Similar to our findings, the presence of closely related Type 3 fimbriae was also demonstrated in *E. coli* and *K. pneumonia* catheter-associated urinary tract infection strains [30], further supporting that fimbriae may be transferred between different genera sharing niche in the same host.

### Only two fimbrial types of *Gallibacterium*, yet only one at once, are expressed *in vitro*

Of the five *Gallibacterium* fimbriae types defined in the present study, evidence for *in vitro* expression of the fimbrial proteins was only available for Flf [15]. In the present study we examined the expression levels of the three fimbrial subunit proteins (FlfA, Flf1A and Flf3A). Western Blot analysis revealed that each antiserum was highly specific for each individual type supporting previous findings that the phylogenetic grouping of the major fimbrial subunits correlates well with the antigenic properties of the proteins [34, 43].

Results from the *in vitro* expression study demonstrated expression of the two of the three fimbrial subunit proteins (FlfA and Flf3A) in the *Gallibacterium* strains investigated. Yet generally, only a single fimbrial subunit protein was expressed at a time in the same strain, suggesting that the expression of *Gallibacterium* fimbriae may be regulated by one of the many fimbrial regulation mechanisms described in other bacteria [56]. Interestingly, the most commonly found fimbriae Flf1A was not expressed in any of 16 *G. anatis* strains encoding the fimbrial cluster. There may be several explanations to this finding: i) as many fimbriae are poorly expressed during laboratory conditions it is likely that the expression of Flf1A requires specific factors only provided by the natural host [18, 19]; ii) highly conserved domain structure belonging to only one CD group ‘a’ observed in Flf1 indicates that these fimbriae likely experience low immune pressure from the host and thus may be a silent or non-functional copy fimbriae of *Gallibacterium* spp.; iii) Flf1 may be important and expressed in the environment outside the host. Based on the whole genome RNA sequencing of *G. anatis* strain UMN179, the *flf1A* gene (UMN179_00750) is transcribed under *in vitro* growth conditions (unpublished results), which suggest that there may be additional processes between the transcription and translation involved in the reduced expression or stability of Flf1A. To reveal the role and expression patterns of the individual fimbrial clusters during *in vitro* and *in vivo* conditions, further investigations, including biologically relevant animal trials, are warranted.

## Conclusions

In this study, the presence of up to three CU fimbrial clusters per individual *Gallibacterium* genome was identified. All fimbrial clusters found in the diverse set of *Gallibacterium* strains shared similar morphology and phylogenetically and were assigned to a single γ_4_ clade of chaperone-usher fimbriae. The major fimbrial subunit and adhesion proteins encoded in *Gallibacterium* genomes displayed high amino acid sequence variation, suggesting that the observed diversity may be maintained through homologous recombination between fimbrial clusters of *Gallibacterium* spp. Fimbriae within a given strain were typically not co-expressed *in vitro*, indicating that different *Gallibacterium* fimbrial types may be expressed during different conditions and may have different role at bacteria-host interaction. Importantly, all *Gallibacterium* fimbriae were systemically classified, and the fimbrial clusters defined in this study allow future research by use of a common framework.

## Methods

### Strains and genome sequences

All genomes used in this study were sequenced and *de novo* assembled as described by Kudirkiene et al. [58]. The genome of *G. anatis* strain 12656/12 Liver is available under the BioProject ID: 213810 [GenBank: AVOX00000000]. The remaining 21 *Gallibacterium* genomes were annotated with the NCBI Prokaryotic Genomes Automatic Annotation Pipeline (http://www.ncbi.nlm.nih.gov/genomes/static/Pipeline.html) and have been deposited at DDBJ/EMBL/GenBank under the BioProject ID: 217951 [GenBank: JPXO00000000, JPXP00000000, JPXQ00000000, JPXR00000000, JPXS00000000, JPXT00000000, JPXU00000000, JPXV00000000, JPXW00000000, JPXX00000000, JPXY00000000, JPHO00000000, JPTU00000000, JPJK00000000, JPJL00000000, JPJM00000000, JPJN00000000, JPJO00000000, JPJP00000000, JPJQ00000000, JPHN00000000]. All annotated genomes were imported into CLC Genomics Workbench v.6.5.1 (CLC, Denmark) for further analysis.

### Detection of Chaperone-Usher (CU) fimbrial clusters

Three fimbrial usher nucleotide sequences (UMN179_00811, UMN179_00293 and UMN179_00752) annotated in *G. anatis* strain UMN179 [14] were used as BLASTn queries against examined *Gallibacterium* genomes. BLASTn searches were performed using CLC Genomics Workbench with default parameters. Ushers with the reported E-value of 0 were selected for further analysis. To enable detection of more variable ushers all genomes were additionally screened for the presence of an usher protein family domain PF00577. Subsequently, the genomic regions encoding all identified ushers were visualized to enable search for other potential fimbrial proteins flanking the genes. Whole clusters containing all fimbrial assembly proteins (major fimbrial protein, chaperone, usher and fimbrial adhesin) along with flanking sequences extended by approximately 300 bp were extracted from the genomes, reversed and complemented if needed, and added to the CLC database.

### Alignments and phylogenetic analysis of CU fimbrial clusters

Nucleotide sequences of the identified CU fimbrial clusters were exported from the database and aligned with the corresponding CU fimbrial clusters UMN179_00809-00812, UMN179_00295-00292 and UMN179_00750-00753 [14] using MAFFT v7.130b [59]. The multiple alignments were viewed using Jalviewv.2.8.0b1 [60] to identify the start and the end positions of the protein encoding sequences for each fimbrial assembly protein in the cluster. Accordingly, the annotations for each gene of the CU fimbrial cluster were corrected to obtain final amino acid sequences for further analyses. After the analysis described below the annotations of the identified chaperone – usher (CU) clusters were edited and resubmitted to GenBank. The locus_tags for the individual fimbrial clusters are listed in Additional file 1: Table S1. Pair-wise comparison of the fimbrial assembly proteins was generated using ClustalOmega [61].

Phylogenetic relationships between the identified *Gallibacterium* CU clusters and CU clusters of other Gram-negative bacteria were inferred using usher amino acid sequences as described by Nuccio and Bäumler [28]. Additionally, amino acid sequences of all four fimbrial assembly proteins (major fimbrial subunit, chaperone, usher and fimbrial adhesin) were used to examine the relationship of CU fimbrial clusters within the genus *Gallibacterium*. The Neighbour-Joining method implemented in the MEGA v.6 software package [62] was used to generate phylogenetic trees. Three previously described UMN179 CU fimbrial clusters [14] were included in the phylogenetic and evolutionary analyses described below.

NCBI Conserved Domain Database (CDD) CDSEARCH/cdd v3.11 tool [63] was used to identify conserved domains in the amino acid sequences of each fimbrial assembly protein of the CU clusters.

### BLASTp

To examine the similarity of fimbrial assembly proteins in genus *Gallibacterium* to the proteins of other bacterial species all amino acid sequences were submitted to BLASTp at NCBI. The sequences were compared to both UniProt/SwissProt and the non-redundant protein databases using default parameters.

### Evolutionary analysis of *Gallibacterium*strains

The inferred evolutionary relationship between strains belonging to *G. anatis* and *G. genomospecies* 1 and 2 was revealed using MLST scheme developed by Johnson et al. [14]. All sequences were retrieved from the sequenced genome deposited in CLC Genomics Workbench using reference sequences of housekeeping locus fragments *(adk, atpD, fumC, gyrB, mdh, recN, infB and thdF)* (http://pubmlst.org/gallibacterium/) as BLASTn queries against the genomes*.* The Maximum Likelihood method based on the Hasegawa-Kishino-Yano model was used to create a phylogenetic tree of the aligned concatenated 8 locus sequences for MLST analysis.

### Western blotting

Western blotting was used to investigate cross-reaction of anti-serum raised against recombinant FlfA, Flf1A (previously Gab_0572) and Flf3A (previously Gab_2156), as well as *in vitro* expression of these proteins. The production of recombinant FlfA, Flf1A and Flf3A is described in Bager *et al*. [39]. FlfA and Flf1A were purified in a soluble form, while Flf3A was purified in an insoluble form. The production of polyclonal antisera against FlfA is described in Bager *et al*. [15]. The production of polyclonal antisera against Flf1Aand Flf3A was carried out as described for FlfA. Rabbit antiserum was collected at day 0 (pre-immune) and day 41 (immune). To investigate *in vitro* expression, whole cell extracts were prepared from *in vitro* cultured cells. In short, 22 *Gallibacterium* strains were incubated in liquid BHI to reach the early-stationary growth phase. Cells were harvested by centrifugation, washed and resuspended in PBS, and lysed by four freeze-thaw cycles. The samples were quantified as described in Bager *et al*. [15]. For the Western blotting, a volume corresponding to an OD_260_-value of 8 of each whole cell extract, or 0.1 μg recombinant protein, was electrophoresed in a 4-12% NuPAGE Bis-Tris gel (Invitrogen) under reducing conditions. The proteins were transferred to a polyvinylidenedifluoride (PVDF) membrane by use of the iBlot dry blotting system (Invitrogen) and blocked overnight in phosphate-buffered saline (PBS) plus 0.1% Tween-20 plus 5% skim milk. The membranes were incubated with pre-immune or immune rabbit antiserum. FlfA anti-serum was diluted 1:4,000 in diluting buffer (PBS plus 0.1% Tween-20 plus 3% skim milk), while Flf1A and Flf3A rabbit antiserum was diluted 1:2,000. Polyclonal goat anti-rabbit IgG (Fc): HRP (AbDSerotec, diluted 1:10,000 in diluting buffer) was used as the secondary antibody. The blots were developed using the Novex® ECL Chemiluminescent Substrate Reagent Kit (Invitrogen).

## Electronic supplementary material

Additional file 1: Table S1: The 48 fimbrial clusters identified in 22 strains of *Gallibacterium* spp. (XLSX 18 KB)

Additional file 2: Figure S1: Evolutionary relationships of chaperone proteins identified in *Gallibacterium* strains. A total of 211 amino acid positions were used to infer the evolutionary relationship of 51 aligned chaperone proteins. Data was analyzed using the Neighbor-Joining method and conducted in MEGA6. Bootstrap values (1000) of more than 90 are displayed next to the branches. The scale represents the number of amino acid substitutions per site computed using the Poisson correction method. Colors at the end of the branches indicate phylogenetic group (FlfA, Flf1A, Flf2A, Flf3A and Flf4A) of the fimbrial subunit protein detected in the same fimbrial cluster as the chaperone under the analysis. (PNG 48 KB)

Additional file 3: Figure S2: Evolutionary relationships of usher proteins identified in *Gallibacterium* strains. A total of 688 amino acid positions were used to infer the evolutionary relationship of 51 aligned usher proteins. Data was analyzed using the Neighbor-Joining method and conducted in MEGA6. Bootstrap values (1000) of more than 90 are displayed next to the branches. The scale represents the number of amino acid substitutions per site computed using the Poisson correction method. Colors at the end of the branches indicate phylogenetic group (FlfA, Flf1A, Flf2A, Flf3A and Flf4A) of the fimbrial subunit protein detected in the same fimbrial cluster as the usher under the analysis. (PNG 47 KB)

Additional file 4: Figure S3: Evolutionary relationships of adhesin proteins identified in *Gallibacterium* strains. A total of 514 amino acid positions were used to infer the evolutionary relationship of 51 aligned adhesin proteins. Data was analyzed using the Neighbor-Joining method and conducted in MEGA6. Bootstrap values (1000) of more than 90 are displayed next to the branches. The scale represents the number of amino acid substitutions per site computed using the Poisson correction method. Colors at the end of the branches indicate phylogenetic group (FlfA, Flf1A, Flf2A, Flf3A and Flf4A) of the fimbrial subunit protein detected in the same fimbrial cluster as the adhesin under the analysis. (PNG 45 KB)

Additional file 5: Table S2: Genetic organization of regions flanking CU fimbrial clusters of *Gallibacterium* spp. Depending on the CU cluster, genes found in the regions from 2600 to 4500 bp upstream and downstream of the fimbrial cluster are shown. Acccession numbers of the genes flanking fimbrial clusters are given as found in strain UMN179. (XLSX 17 KB)

Additional file 6: Figure S4: Evolutionary relationship of usher sequences from *Gallibacterium* and from different Gram-negative bacteria. The analysis involved 239 amino acid sequences. There were a total of 99 positions in the final dataset. Data was analyzed using the Neighbor-Joining method and conducted in MEGA6. The evolutionary distances were computed using the JTT matrix-based method and are in the units of the number of amino acid substitutions per site. Nuccio clade γ_4_ is shown with the blue color and ushers of *Gallibacterium* spp. are shown in green. (PNG 174 KB)

Additional file 7: Table S3: The similarity of CU fimbrial proteins of *Gallibacterium* spp. to the proteins in the non-redundunt protein database. The first column shows CU fimbrial type determined based on the major fimbrial subunit protein phylogeny. The range in percent indicates the similarity of the particular protein to the proteins in the database depending on the strain analyzed. The coverage of the protein is shown in the brackets. (XLSX 11 KB)

Additional file 8: Figure S5: Evolutionary relationships of *Gallibacterium* and other bacterium species using partial sequence of 16sRNA gene. The analysis involved 13 nucleotide sequences. The strains used in the analysis are designated at the end of the branches indicate. A total of 1256 positions were in the final dataset. The evolutionary history was inferred using the Neighbor-Joining method and conducted in MEGA6. The evolutionary distances were computed using the Jukes-Cantor method and are in the units of the number of base substitutions per site. The percentage of replicate trees in which the associated taxa clustered together in the bootstrap test (1000 replicates) are shown next to the branches. (PNG 22 KB)

Additional file 9: Figure S6: Evolutionary relationship of the fimbrial subunit proteins from *Gallibacterium* and from different bacteria. A total of 150 amino acid positions were used to infer the evolutionary relationship of 62 aligned fimbrial subunit proteins. Data was analyzed using the Neighbor-Joining method and conducted in MEGA6. Bootstrap values (1000) of more than 90 are displayed next to the branches. The scale represents the number of amino acid substitutions per site computed using the Poisson correction method. Fimbrial subunit proteins phylogenetic groups (FlfA, Flf1A, Flf2A, Flf3A and Flf4A) defined among fimbrial clusters in *Gallibacterium* strains are shown in different colors. (PNG 61 KB)

## References

[1] Bojesen AM, Nielsen SS, Bisgaard M (2003). Prevalence and transmission of haemolytic *Gallibacterium* species in chicken production systems with different biosecurity levels. Avian Pathol.

[2] Bojesen AM, Nielsen OL, Christensen JP, Bisgaard M (2004). *In vivo* studies of *Gallibacterium anatis* infection in chickens. Avian Pathol.

[3] Neubauer C, De Souza-Pilz M, Bojesen AM, Bisgaard M, Hess M (2009). Tissue distribution of haemolytic *Gallibacterium anatis* isolates in laying birds with reproductive disorders. Avian Pathol.

[4] Zepeda VA, Calderón-Apodaca NL, Paasch ML, Martín PG, Paredes DA, Ramírez-Apolinar S, Soriano-Vargas E (2010). Histopathologic findings in chickens experimentally infected with *Gallibacterium anatis* by nasal instillation. Avian Dis.

[5] Jones KH, Thornton JK, Zhang Y, Mauel MJ (2013). A 5-year retrospective report of *Gallibacterium anatis* and *Pasteurella multocida* isolates from chickens in Mississippi. Poult Sci.

[6] Paudel S, Alispahic M, Liebhart D, Hess M, Hess C (2013). Assessing pathogenicity of *Gallibacterium anatis* in a natural infection model: the respiratory and reproductive tracts of chickens are targets for bacterial colonization. Avian Pathol.

[7] Bojesen AM, Shivaprasad HL (2007). Genetic diversity of *Gallibacterium* isolates from California turkeys. Avian Pathol.

[8] Christensen H, Bisgaard M, Bojesen AM, Mutters R, Olsen JE (2003). Genetic relationships among avian isolates classified as *Pasteurella haemolytica*, *‘Actinobacillus salpingitidis’* or *Pasteurella anatis* with proposal of *Gallibacterium anatis* gen. *nov*., comb. *nov*. and description of additional genomospecies within *Gallibacterium* gen. *nov*. Int J Syst Evol Microbiol.

[9] Bisgaard M, Korczak BM, Busse HJ, Kuhnert P, Bojesen AM, Christensen H (2009). Classification of the taxon 2 and taxon 3 complex of Bisgaard within *Gallibacterium* and description of *Gallibacterium melopsittaci* sp. *nov*., *Gallibacterium trehalosifermentans* sp. *nov*. and *Gallibacterium salpingitidis* sp. *nov*. Int J Syst Evol Microbiol.

[10] Kristensen BM, Frees D, Bojesen AM (2010). GtxA from *Gallibacterium anatis*, a cytolytic RTX-toxin with a novel domain organisation. Vet Res.

[11] Kristensen BM, Frees D, Bojesen AM (2011). Expression and secretion of the RTX-toxin GtxA among members of the genus *Gallibacterium*. Vet Microbiol.

[12] García-Gómez E, Vaca S, Pérez-Méndez A, Ibarra-Caballero J, Pérez-Márquez V, Tenorio VR, Negrete-Abascal E (2005). *Gallibacterium anatis*-secreted metalloproteases degrade chicken IgG. Avian Pathol.

[13] Lucio MLS, Vaca S, Vázquez C, Zenteno E, Rea I, Pérez-Márquez VM, Negrete-Abascal E (2012). Adhesion of *Gallibacterium anatis* to chicken oropharyngeal epithelial cells and the identification of putative fimbriae. Adv Microbiol.

[14] Johnson TJ, Danzeisen JL, Trampel D, Nolan LK, Seemann T, Bager RJ, Bojesen AM (2013). Genome analysis and phylogenetic relatedness of *Gallibacterium anatis* strains from poultry. PLoS One.

[15] Bager RJ, Nesta B, Pors SE, Soriani M, Serino L, Boyce JD, Adler B, Bojesen AM (2013). The fimbrial protein FlfA from *Gallibacterium anatis* is a virulence factor and vaccine candidate. Infect Immun.

[16] Hung DL, Knight SD, Woods RM, Pinkner JS, Hultgren SJ (1996). Molecular basis of two subfamilies of immunoglobulin-like chaperones. EMBO J.

[17] Klemm P, Schembri MA (2000). Fimbrial surface display systems in bacteria: from vaccines to random libraries. Microbiology.

[18] Humphries AD, Raffatellu M, Winter S, Weening EH, Kingsley RA, Droleskey R, Zhang S, Figueiredo J, Khare S, Nunes J, Adams LG, Tsolis RM, Bäumler AJ (2003). The use of flow cytometry to detect expression of subunits encoded by 11 *Salmonella enterica* serotype *Typhimurium* fimbrial operons. Mol Microbiol.

[19] Low AS, Holden N, Rosser T, Roe AJ, Constantinidou C, Hobman JL, Smith DG, Low JC, Gally DL (2006). Analysis of fimbrial gene clusters and their expression in enterohaemorrhagic *Escherichia coli* O157:H7. Environ Microbiol.

[20] Gohl O, Friedrich A, Hoppert M, Averhoff B (2006). The thin pili of *Acinetobacter* sp. strain BD413 mediate adhesion to biotic and abiotic surfaces. Appl Environ Microbiol.

[21] Rocha SP, Pelayo JS, Elias WP (2007). Fimbriae of uropathogenic *Proteus mirabilis*. FEMS Immunol Med Microbiol.

[22] Korea CG, Badouraly R, Prevost MC, Ghigo JM, Beloin C (2010). *Escherichia coli* K-12 possesses multiple cryptic but functional chaperone-usher fimbriae with distinct surface specificities. Environ Microbiol.

[23] Korea CG, Ghigo JM, Beloin C (2011). The sweet connection: Solving the riddle of multiple sugar-binding fimbrial adhesins in Escherichia coli: multiple E. coli fimbriae form a versatile arsenal of sugar-binding lectins potentially involved in surface-colonisation and tissue tropism. Bioessays.

[24] van Aartsen JJ, Stahlhut SG, Harrison EM, Crosatti M, Ou HY, Krogfelt KA, Struve C, Rajakumar K (2012). Characterization of a novel chaperone/usher fimbrial operon present on KpGI-5, a methionine tRNA gene-associated genomic island in *Klebsiella pneumoniae*. BMC Microbiol.

[25] Wang C, Hu YH, Chi H, Sun L (2013). The major fimbrial subunit protein of *Edwardsiella tarda*: vaccine potential, adjuvant effect, and involvement in host infection. Fish Shellfish Immunol.

[26] Wurpel DJ, Beatson SA, Totsika M, Petty NK, Schembri MA (2013). Chaperone-usher fimbriae of *Escherichia coli*. PLoS One.

[27] Kuan L, Schaffer J, Zouzias CD, Pearson MM (2014). Characterization of 17 chaperone-usher fimbriae encoded by *Proteus mirabilis* reveals strong conservation. J Med Microbiol.

[28] Nuccio SP, Bäumler AJ (2007). Evolution of the chaperone/usher assembly pathway: fimbrial classification goes Greek. Microbiol Mol Biol Rev.

[29] Allen BL, Gerlach GF, Clegg S (1991). Nucleotide sequence and functions of mrk determinants necessary for expression of type 3 fimbriae in *Klebsiella pneumoniae*. J Bacteriol.

[30] Ong CL, Beatson SA, Totsika M, Forestier C, McEwan AG, Schembri MA (2010). Molecular analysis of type 3 fimbrial genes from *Escherichia coli, Klebsiella and Citrobacter species*. BMC Microbiol.

[31] Scavone P, Umpiérrez A, Maskell DJ, Zunino P (2011). Nasal immunization with attenuated *Salmonella* Typhimurium expressing an MrpA-TetC fusion protein significantly reduces *Proteus mirabilis* colonization in the mouse urinary tract. J Med Microbiol.

[32] Gaastra W, Svennerholm AM (1996). Colonization factors of human enterotoxigenic *Escherichia coli* (ETEC). Trends Microbiol.

[33] Pellegrino R, Galvalisi U, Scavone P, Sosa V, Zunino P (2003). Evaluation of *Proteus mirabilis* structural fimbrial proteins as antigens against urinary tract infections. FEMS Immunol Med Microbiol.

[34] Anantha RP, McVeigh AL, Lee LH, Agnew MK, Cassels FJ, Scott DA, Whittam TS, Savarino SJ (2004). Evolutionary and functional relationships of colonization factor antigen i and other class 5 adhesive fimbriae of enterotoxigenic *Escherichia coli*. Infect Immun.

[35] Tiels P, Verdonck F, Coddens A, Goddeeris B, Cox E (2008). The excretion of F18+ E. coli is reduced after oral immunisation of pigs with a FedF and F4 fimbriae conjugate. Vaccine.

[36] Ruan X, Liu M, Casey TA, Zhang W (2011). A tripartite fusion, FaeG-FedF-LT(192)A2:B, of enterotoxigenic *Escherichia coli* (ETEC) elicits antibodies that neutralize cholera toxin, inhibit adherence of K88 (F4) and F18 fimbriae, and protect pigs against K88ac/heat-labile toxin infection. Clin Vaccine Immunol.

[37] Sadilkova L, Nepereny J, Vrzal V, Sebo P, Osicka R (2012). Type IV fimbrial subunit protein ApfA contributes to protection against porcine pleuropneumonia. Vet Res.

[38] Hur J, Lee JH (2012). Development of a novel live vaccine delivering enterotoxigenic *Escherichia coli* fimbrial antigens to prevent post-weaning diarrhea in piglets. Vet Immunol Immunopathol.

[39] Bager RJ, Kudirkiene E, da Piedade I, Seemann T, Nielsen TK, Pors SE, Mattsson AH, Boyce JD, Adler B, Bojesen AM (2014). *In silico* prediction of *Gallibacterium anatis* pan-immunogens. Vet Research.

[40] Bojesen AM, Torpdahl M, Christensen H, Olsen JE, Bisgaard M (2003). Genetic diversity of *Gallibacterium anatis* isolates from different chicken flocks. J Clin Microbiol.

[41] Lawrence JG, Retchless AC (2009). The interplay of homologous recombination and horizontal gene transfer in bacterial speciation. Methods Mol Biol.

[42] Lintermans PF, Bertels A, Schlicker C, Deboeck F, Charlier G, Pohl P, Norgren M, Normark S, van Montagu M, De Greve H (1991). Identification, characterization, and nucleotide sequence of the F17-G gene, which determines receptor binding of *Escherichia coli* F17 fimbriae. J Bacteriol.

[43] Bertin Y, Girardeau JP, Darfeuille-Michaud A, Contrepois M (1996). Characterization of 20 K fimbria, a new adhesin of septicemic and diarrhea-associated *Escherichia coli* strains, that belongs to a family of adhesins with N-acetyl-D-glucosamine recognition. Infect Immun.

[44] Clermont O, Olier M, Hoede C, Diancourt L, Brisse S, Keroudean M, Glodt J, Picard B, Oswald E, Denamur E (2011). Animal and human pathogenic *Escherichia coli* strains share common genetic backgrounds. Infect Genet Evol.

[45] Townsend SM, Kramer NE, Edwards R, Baker S, Hamlin N, Simmonds M, Stevens K, Maloy S, Parkhill J, Dougan G, Bäumler AJ (2001). *Salmonella enterica* serovar Typhi possesses a unique repertoire of fimbrial gene sequences. Infect Immun.

[46] Martin C, Rousset E, De Greve H (1997). Human uropathogenic and bovine septicaemic *Escherichia coli* strains carry an identical F17-related adhesin. Res Microbiol.

[47] Cid D, Sanz R, Marín I, de Greve H, Ruiz-Santa-Quiteria JA, Amils R, de la Fuente R (1999). Characterization of nonenterotoxigenic *Escherichia coli* strains producing F17 fimbriae isolated from diarrheic lambs and goat kids. J Clin Microbiol.

[48] Moch T, Hoschützky H, Hacker J, Kröncke KD, Jann K (1987). Isolation and characterization of the alpha-sialyl-beta-2,3-galactosyl-specific adhesin from fimbriated *Escherichia coli*. Proc Natl Acad Sci U S A.

[49] Tanskanen J, Saarela S, Tankka S, Kalkkinen N, Rhen M, Korhonen TK, Westerlund-Wikström B (2001). The *gaf* fimbrial gene cluster of *Escherichia coli* expresses a full-size and a truncated soluble adhesin protein. J Bacteriol.

[50] Cahoon LA, Seifert HS (2011). Focusing homologous recombination: pilin antigenic variation in the pathogenic *Neisseria*. Mol Microbiol.

[51] Kong Y, Ma JH, Warren K, Tsang RS, Low DE, Jamieson FB, Alexander DC, Hao W (2013). Homologous recombination drives both sequence diversity and gene content variation in *Neisseria meningitidis*. Genome Biol Evol.

[52] Forslund K, Pekkari I, Sonnhammer EL (2011). Domain architecture conservation in orthologs. BMC Bioinformatics.

[53] Read TD, Dowdell M, Satola SW, Farley MM (1996). Duplication of pilus gene complexes of *Haemophilus influenzae* biogroup *aegyptius*. J Bacteriol.

[54] Holden NJ, Gally DL (2004). Switches, cross-talk and memory in *Escherichia coli* adherence. J Med Microbiol.

[55] Lucchini S, Rowley G, Goldberg MD, Hurd D, Harrison M, Hinton JC (2006). H-NS mediates the silencing of laterally acquired genes in bacteria. PLoS Pathog.

[56] Clegg S, Wilson J, Johnson J (2011). More than one way to control hair growth: regulatory mechanisms in enterobacteria that affect fimbriae assembled by the chaperone/usher pathway. J Bacteriol.

[57] Aguilar-Bultet L, Calderon-Copete SP, Frey J, Falquet L (2013). Draft genome sequence of the virulent *Avibacterium paragallinarum* Serotype A strain JF4211 and identification of two toxins. Genome Announc.

[58] Kudirkiene E, Christensen H, Bojesen AM (2013). Draft genome sequence of *Gallibacterium anatis* bv. *haemolytica* 12656–12 liver, an isolate obtained from the liver of a septicemic chicken. Genome Announc.

[59] Katoh K, Standley DM (2013). MAFFT multiple sequence alignment software version 7: improvements in performance and usability. Mol Biol Evol.

[60] Waterhouse AM, Procter JB, Martin DM, Clamp M, Barton GJ (2009). Jalview version 2–a multiple sequence alignment editor and analysis workbench. Bioinformatics.

[61] Goujon M, McWilliam H, Li W, Valentin F, Squizzato S, Paern J, Lopez R (2010). A new bioinformatics analysis tools framework at EMBL-EBI. Nucleic Acids Res.

[62] Tamura K, Stecher G, Peterson D, Filipski A, Kumar S (2013). MEGA6: Molecular Evolutionary Genetics Analysis version 6.0. Mol Biol Evol.

[63] Marchler-Bauer A, Lu S, Anderson JB, Chitsaz F, Derbyshire MK, DeWeese-Scott C, Fong JH, Geer LY, Geer RC, Gonzales NR, Gwadz M, Hurwitz DI, Jackson JD, Ke Z, Lanczycki CJ, Lu F, Marchler GH, Mullokandov M, Omelchenko MV, Robertson CL, Song JS, Thanki N, Yamashita RA, Zhang D, Zhang N, Zheng C, Bryant SH (2011). CDD: a Conserved Domain Database for the functional annotation of proteins. Nucleic Acids Res.

