# Supplementary figures and images for: Chaperone-usher fimbriae in a diverse selection of Gallibacterium genomes
Source: BMC Genomics. 2014 Dec 12;15(1):1093. doi: 10.1186/1471-2164-15-1093 (PMC4299563; doi:10.1186/1471-2164-15-1093)

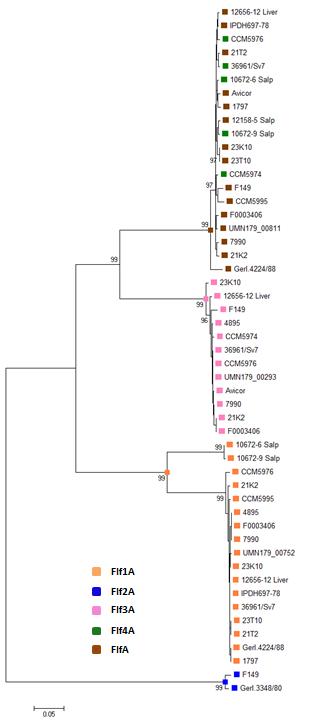

Supplement: Supplementary file 2 — Additional file 2: Figure S1: Evolutionary relationships of chaperone proteins identified in Gallibacterium strains. A total of 211 amino acid positions were used to infer the evolutionary relationship of 51 aligned chaperone proteins. Data was analyzed using the Neighbor-Joining method and conducted in MEGA6. Bootstrap values (1000) of more than 90 are displayed next to the branches. The scale represents the number of amino acid substitutions per site computed using the Poisson correction method. Colors at the end of the branches indicate phylogenetic group (FlfA, Flf1A, Flf2A, Flf3A and Flf4A) of the fimbrial subunit protein detected in the same fimbrial cluster as the chaperone under the analysis. (PNG 48 KB) [file 12864_2014_6899_MOESM2_ESM.png]

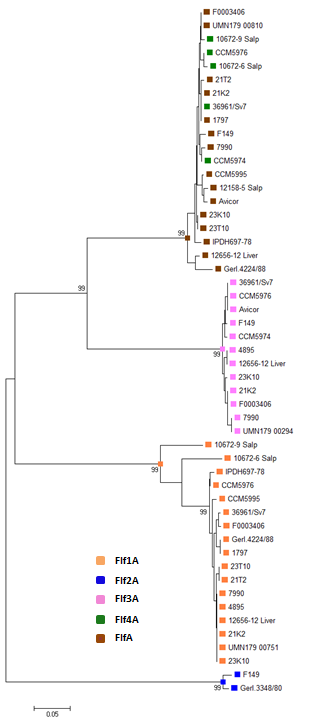

Supplement: Supplementary file 3 — Additional file 3: Figure S2: Evolutionary relationships of usher proteins identified in Gallibacterium strains. A total of 688 amino acid positions were used to infer the evolutionary relationship of 51 aligned usher proteins. Data was analyzed using the Neighbor-Joining method and conducted in MEGA6. Bootstrap values (1000) of more than 90 are displayed next to the branches. The scale represents the number of amino acid substitutions per site computed using the Poisson correction method. Colors at the end of the branches indicate phylogenetic group (FlfA, Flf1A, Flf2A, Flf3A and Flf4A) of the fimbrial subunit protein detected in the same fimbrial cluster as the usher under the analysis. (PNG 47 KB) [file 12864_2014_6899_MOESM3_ESM.png]

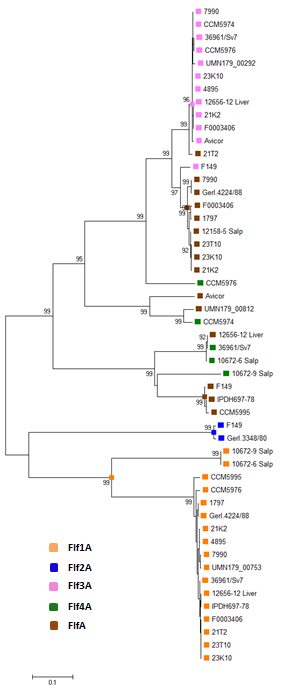

Supplement: Supplementary file 4 — Additional file 4: Figure S3: Evolutionary relationships of adhesin proteins identified in Gallibacterium strains. A total of 514 amino acid positions were used to infer the evolutionary relationship of 51 aligned adhesin proteins. Data was analyzed using the Neighbor-Joining method and conducted in MEGA6. Bootstrap values (1000) of more than 90 are displayed next to the branches. The scale represents the number of amino acid substitutions per site computed using the Poisson correction method. Colors at the end of the branches indicate phylogenetic group (FlfA, Flf1A, Flf2A, Flf3A and Flf4A) of the fimbrial subunit protein detected in the same fimbrial cluster as the adhesin under the analysis. (PNG 45 KB) [file 12864_2014_6899_MOESM4_ESM.png]

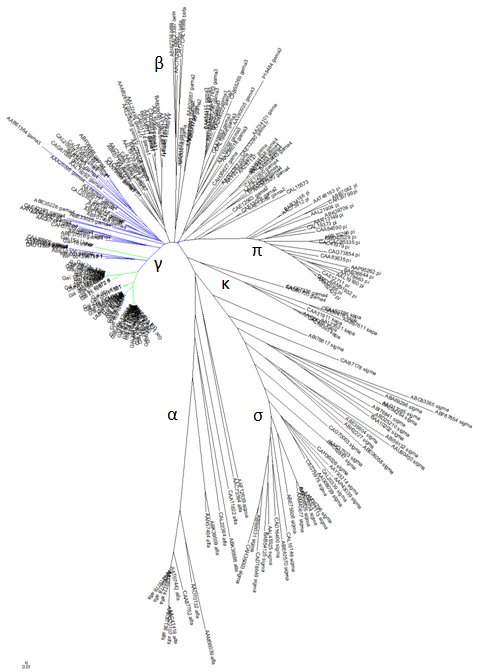

Supplement: Supplementary file 6 — Additional file 6: Figure S4: Evolutionary relationship of usher sequences from Gallibacterium and from different Gram-negative bacteria. The analysis involved 239 amino acid sequences. There were a total of 99 positions in the final dataset. Data was analyzed using the Neighbor-Joining method and conducted in MEGA6. The evolutionary distances were computed using the JTT matrix-based method and are in the units of the number of amino acid substitutions per site. Nuccio clade γ4 is shown with the blue color and ushers of Gallibacterium spp. are shown in green. (PNG 174 KB) [file 12864_2014_6899_MOESM6_ESM.png]

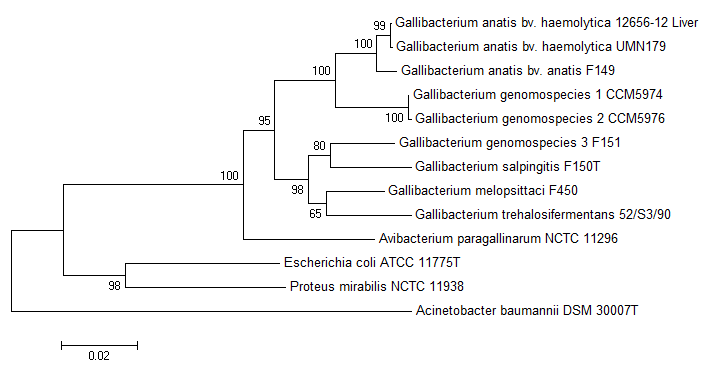

Supplement: Supplementary file 8 — Additional file 8: Figure S5: Evolutionary relationships of Gallibacterium and other bacterium species using partial sequence of 16sRNA gene. The analysis involved 13 nucleotide sequences. The strains used in the analysis are designated at the end of the branches indicate. A total of 1256 positions were in the final dataset. The evolutionary history was inferred using the Neighbor-Joining method and conducted in MEGA6. The evolutionary distances were computed using the Jukes-Cantor method and are in the units of the number of base substitutions per site. The percentage of replicate trees in which the associated taxa clustered together in the bootstrap test (1000 replicates) are shown next to the branches. (PNG 22 KB) [file 12864_2014_6899_MOESM8_ESM.png]

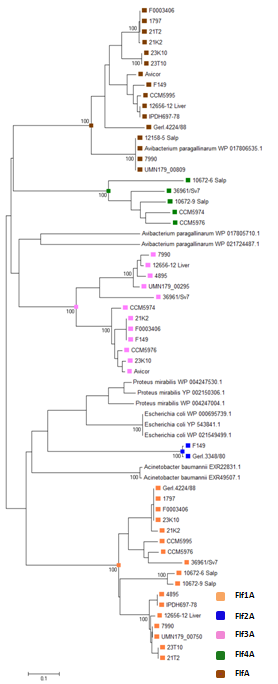

Supplement: Supplementary file 9 — Additional file 9: Figure S6: Evolutionary relationship of the fimbrial subunit proteins from Gallibacterium and from different bacteria. A total of 150 amino acid positions were used to infer the evolutionary relationship of 62 aligned fimbrial subunit proteins. Data was analyzed using the Neighbor-Joining method and conducted in MEGA6. Bootstrap values (1000) of more than 90 are displayed next to the branches. The scale represents the number of amino acid substitutions per site computed using the Poisson correction method. Fimbrial subunit proteins phylogenetic groups (FlfA, Flf1A, Flf2A, Flf3A and Flf4A) defined among fimbrial clusters in Gallibacterium strains are shown in different colors. (PNG 61 KB) [file 12864_2014_6899_MOESM9_ESM.png]
